# Chronic Lithium Intoxication: A Challenging Diagnosis

**DOI:** 10.7759/cureus.52626

**Published:** 2024-01-20

**Authors:** Sofia Ferreira, Sara Santos, Sérgio Gomes Ferreira, Luís Fernandes, Penélope Almeida

**Affiliations:** 1 Internal Medicine, Hospital São Sebastião, Centro Hospitalar de Entre o Douro e Vouga, Santa Maria da Feira, PRT; 2 Internal Medicine, Centro Hospitalar de Entre o Douro e Vouga, Santa Maria da Feira, PRT

**Keywords:** chronic renal insufficiency, diabetes insipidus nephrogenic, toxicity, bipolar disorder, lithium carbonate

## Abstract

Lithium has been used in clinical practice since the 1970s. This medication is commonly used to treat and prevent bipolar disorder, but it has a narrow therapeutic index, making toxicity a frequent occurrence. Chronic lithium intoxication can arise due to progressive accumulation, particularly in contexts of dehydration. The effects of chronic lithium intoxication on the nervous, renal, and cardiac systems, as well as on the thyroid and parathyroid glands, are well documented in the literature. The authors present the case of a 66-year-old woman with schizoaffective psychosis and chronic kidney disease, admitted due to altered mental status and dysarthria. Notwithstanding an earlier clinical recommendation to cease lithium administration more than a year ago, the patient continued its usage, culminating in neurological, cardiac, renal, and endocrine manifestations. Although the diagnosis was delayed, her clinical progression was favorable, obviating the need for renal replacement therapy. This case highlights the importance of a detailed medical history and the diagnostic challenges in clinical practice. The use of this drug without proper monitoring can lead to multisystem dysfunction.

## Introduction

After decades of use, lithium carbonate continues to be considered the first-line therapy for the treatment and prevention of bipolar and other psychiatric disorders [1, 2].

In 1949, John Frederick Joseph Cade tested the calming effects of lithium on 10 manic patients, achieving clinical stability in all of them [3]. However, in that same year, several fatal cases associated with lithium poisoning were reported, leading to the drug being withdrawn from the American market. The US FDA approved this drug for treating mood disorders in 1970, but only after the capability to monitor its serum levels became available [4,5].

Lithium poisoning can be acute, due to the accidental or intentional ingestion of excessive amounts of lithium; acute-on-chronic, when a patient treated with lithium ingests a large number of tablets at once; or chronic, as a result of deteriorating kidney function. Unlike acute lithium poisoning, chronic poisoning occurs gradually over an extended period. The symptoms and signs of toxicity differ based on the total body burden of lithium and the rate of onset of toxicity. Chronic lithium poisoning may lead to a range of symptoms and signs that can include neurological, gastrointestinal, and renal manifestations [4]. Acute and chronic lithium toxicity present distinct clinical profiles, primarily differing in the timing and prominence of specific symptoms. In chronic toxicity, the onset is gradual, and neurologic signs and symptoms tend to predominate early in the course of the condition. It's important to note that chronic lithium poisoning may be challenging to detect early on, as symptoms can be subtle and nonspecific. In contrast, acute lithium toxicity is characterized by a more abrupt onset, with gastrointestinal symptoms like nausea, vomiting, and diarrhea being the primary features. Notably, neurologic symptoms in acute toxicity may have a delayed presentation [4].

## Case presentation

The patient was a 66-year-old woman with a medical history of schizoaffective psychosis and chronic kidney disease stage 4 (G4A1) by Kidney Disease Improving Global Outcomes (KDIGO) criteria [6] of unknown etiology. She was followed in a psychiatry outpatient clinic and, according to clinical records, was prescribed quetiapine and lorazepam; however, the ongoing therapy could not be confirmed upon admission. She was brought to the emergency department due to altered consciousness and language disturbances that had been progressing for a week. She denied any recent complications or cardiopulmonary, gastrointestinal, or genitourinary symptoms.

On physical examination, she appeared prostrate (scoring nine on the Glasgow Coma Scale, E3V2M4), pale, and dehydrated; afebrile, normotensive, and with a normal heart rate. She exhibited perioral fasciculations, dysarthria, flaccid tetraparesis, hyperreflexia, and bilateral clonus. Ocular motility was unremarkable without nystagmus. No muscular strength or sensory asymmetries were noted, with a flexor plantar reflex bilaterally.

Among the diagnostic tests conducted upon admission, acute kidney injury (KDIGO stage 2) was evident [7], with an estimated glomerular filtration rate (eGFR) of 11 mL/min/1.73 m2, hypernatremia with serum hyperosmolality, and hypothyroidism, as evidenced in Table 1.

**Table 1:** Laboratory results upon the patient's admission

Laboratory parameter	Patient's value	Reference range
White blood cell	7.0 x10^9^/L	4.0 – 11.0 x10^9^/L
C-reactive protein	1.3 mg/L	< 5,0 mg/L
Creatinine	4.1 mg/dL	0.6 – 1.1 mg/dL
Urea	204 mg/dL	15 - 45 mg/dL
Sodium	149 mmol/L	135 - 145 mmol/L
Potassium	6.4 mmol/L	3.5 – 5.1 mmol/L
Serum osmolality	390 mOsm/Kg	275 – 295 mOsm/Kg
Thyroid-stimulating hormone	5.13 µUI/mL	0.35 – 4.94 µUI/mL
Free thyroxine	5.8 pmol/L	9 - 19 pmol/L
Urinalysis		
pH	6.0	4.8 – 7.40
Density	1.015	1.003 – 1.030
Leukocytes	Negative	< 10
Nitrites	Negative	-
Proteins	Negative	< 10
Erythrocytes	Negative	0-5/µL
Protein-to-creatinine ratio (spot urine sample)	0.09 mg/mg	< 0.2 mg/mg
Urinary osmolality	518 mOsm/Kg	500 to 850 mOsm/kg
Urinary sodium	49 mmol/L	< 20 mmol/L

The urinalysis revealed no significant alterations (Table 1). A 12-lead electrocardiogram (ECG) showed sinus rhythm with a heart rate of 82 beats/minute, peaked T-waves, and a prolonged QTc interval of 534 ms. Renal-bladder ultrasound ruled out obstructive pathology, and a cranial CT scan showed no acute vascular pathology or space-occupying lesions.

She was admitted to the internal medicine department for further evaluation and treatment, with psychotropic medications discontinued and intravenous fluid therapy initiated. During hospitalization, there was progressive improvement in renal function with fluid therapy; however, sustained polyuria with hypernatremia was observed. On the fourth day of admission, after contacting a day center assisting with the patient's medication management, it was discovered that she had been taking lithium carbonate, which should have been discontinued based on the assistant psychiatrist's recommendation over a year ago. A plasma lithium level was then measured, which was still close to therapeutic limits (lithium level of 0.6 mmol/L), even after five days of isotonic fluid therapy. The neurological, cardiological, and renal manifestations and fluid-electrolyte balance were consistent with chronic lithium intoxication.

From a renal perspective, there was polyuria with intermediate urinary osmolality (518 mOsm/kg on admission), improvement in renal dysfunction, and sustained hypernatremia in a patient without preserved thirst reflex after intensive fluid therapy. A mixed mechanism is considered likely, involving osmotic diuresis (uremia) and partial nephrogenic diabetes insipidus.

Neurological manifestations, characterized by encephalopathy with signs of both first and second neurons, are attributable to chronic lithium intoxication. Importantly, other acute neurological pathologies were ruled out, as the patient underwent cranial and cervical MRI and cerebrospinal fluid examination.

Additionally, the electrocardiographic changes and hypothyroidism were also attributable to this clinical picture.

The patient showed progressive clinical improvement, with a recovery of neurological status and significant improvement in functional performance with support from physical medicine and rehabilitation. Concurrently, there was normalization of renal function with intensified fluid therapy for positive fluid balance and discontinuation of the offending drug. It is worth noting that fluid therapy was halted on the twelfth day of admission, with no observed lithium rebound phenomenon, which can sometimes occur due to transcellular movement and slow drug diffusion [8].

## Discussion

The patient had been on lithium medication for over six years, despite her psychiatrist's recommendation to discontinue due to progressive renal function deterioration. The persistence of this drug led to severe chronic intoxication, with initial lithium levels unknown due to the inability to clarify ongoing therapy.

Chronic lithium toxicity, which we will delve into, presents differently from acute or acute-on-chronic toxicity [4]. Prolonged lithium use induces nephrogenic diabetes insipidus (NDI) in about 20% of consumers. This condition is characterized by renal resistance to the action of vasopressin (anti-diuretic hormone (ADH)), leading to reduced urine concentration ability, resulting in polyuria with diluted urine excretion. Typically, increased thirst (polydipsia) and subsequent increased water intake compensate for these fluid losses. However, when other factors interfere, such as a suppressed thirst reflex or physical incapacity preventing free water intake, dehydration with intravascular volume depletion and toxicity development can occur [9]. Additionally, certain drugs, like non-steroidal anti-inflammatory drugs or angiotensin-converting enzyme inhibitors, may decrease lithium excretion, elevating its serum concentration to potentially toxic levels.

Clinically, besides the aforementioned renal effects (which are both a consequence and a cause of subsequent toxicity), chronic lithium intoxication is predominantly characterized by neurological findings [4]. The chronic nature of the drug increases the risk of neurotoxicity due to prolonged exposure, facilitating accumulation in neural tissue, and the drug's extended half-life due to altered renal excretion and reabsorption. In this context, lithium plasma concentration better correlates with central nervous system levels, and patients may show toxicity signs even with serum levels near the therapeutic range.

Mild toxicity symptoms include tremors, agitation, hyperreflexia, and muscle weakness, typically occurring when lithium levels range between 1.5 and 2.5 mmol/L. Moderate-severity clinical signs such as stupor, rigidity, hypertonia, and clonus can manifest when levels are between 2.5 and 3.5 mmol/L. Severe toxicity is generally observed when lithium levels exceed 3.5 mmol/L, leading to myoclonus, seizures, non-convulsive status epilepticus, and coma [4,10,11].

From a cardiac standpoint, lithium toxicity can result in ECG alterations like prolonged QTc interval, T-wave inversion, flattening, and bradycardia. However, severe cardiac events or elevated cardiac biomarkers are rare with lithium intoxication [11-15]. Additionally, both thyroid gland disorders (mainly hypothyroidism but also hyperthyroidism) and secondary hypercalcemia due to hyperparathyroidism have been reported with lithium poisoning [4,13,16].

The diagnosis of chronic lithium toxicity is primarily based on clinical signs and confirmed by serum lithium concentration [4]. Serum lithium levels help evaluate toxicity severity and determine the need for hemodialysis. While therapeutic levels range between 0.4 and 1.0 mmol/L, initial levels can confirm exposure but might not represent peak serum concentrations in patients on sustained-release formulations. Thus, monitoring should be conducted with increased frequency during the initial phase and subsequently at six-month intervals thereafter. Enhanced monitoring is imperative for individuals prescribed interacting drugs, the elderly, and those exhibiting established renal impairment or other relevant physical illness [17, 18]. Importantly, lithium levels often don't correlate with clinical signs, especially in acute ingestions where serum levels above 4 mmol/L might not always reflect symptoms due to slow central nervous system absorption. Treatment decisions should therefore rely on clinical manifestations rather than drug levels [4, 19].

## Conclusions

In conclusion, while lithium carbonate remains a cornerstone treatment for bipolar and other psychiatric disorders, this case highlights the serious risks associated with its unmonitored and prolonged use, particularly in patients with underlying renal impairment. The multifaceted clinical manifestations observed emphasize the complex consequences of chronic lithium intoxication, underscoring the need for rigorous patient monitoring, regular drug regimen evaluations, and timely diagnostic interventions.
